# Potassium Iodide Tablets Instead of a Saturated Solution Preoperatively for Reaching Euthyroidism Quickly in Refractory Graves’ Disease

**DOI:** 10.7759/cureus.15854

**Published:** 2021-06-23

**Authors:** Costanza Chiapponi, Matthias Schmidt, Michael Faust

**Affiliations:** 1 General, Abdominal, Tumor and Transplant Surgery, University Hospital of Cologne, Cologne, DEU; 2 Endocrine Surgery, Evangelisches Klinikum Köln Weyertal, Cologne, DEU; 3 Clinic of Nuclear Medicine, University Hospital of Cologne, Cologne, DEU; 4 Polyclinic for Endocrinology, Diabetes and Preventive Medicine, University Hospital of Cologne, Cologne, DEU

**Keywords:** graves´disease, potassium iodide, euthyroidism, thyroid surgery, thyreostatic therapy

## Abstract

The optional use of a saturated solution of potassium iodide in the immediate preoperative period to reach euthyroidism is included both in the American Thyroid Association (ATA) and in the European Thyroid Association (ETA) guidelines for the treatment of Graves’ disease (GD). The recent literature though, shows that it does not translate to more clinically meaningful differences in surgical outcome. In our experience, potassium iodide should not be seen as a means for reducing operative time or complications; it is an effective way for reaching euthyroidism quickly. Herein, we describe three selected cases in which concentrated potassium iodide 65 mg tablets - instead of a saturated solution - were administered for thyroid blocking preoperatively, as recommended in the event of a nuclear emergency. One of the patients was pregnant. After oral treatment with potassium iodide 130 mg daily (two pills), euthyroidism was reached in all three cases within 24 hours. There were no side effects and surgery was performed without complications. Although the current literature did not report a significant benefit concerning operative time and complications, in our opinion preoperative potassium iodide plays an important role in selected cases for reaching euthyroidism preoperatively quickly. Potassium iodide 65 mg tablets, which are recommended in case of a nuclear emergency, are a very effective alternative to saturated solutions, which are not always quickly available and generally need to be administered over seven to 10 days.

## Introduction

Although the American Thyroid Association (ATA) and the European Thyroid Association (ETA) guidelines for Graves’ disease (GD) include the use of a saturated solution of potassium iodide (SSKI) in the immediate preoperative period for reaching euthyroidism and reducing thyroid hypervascularity [1,2], the current literature suggests that it does not translate to more clinically meaningful differences in surgical outcome and questions its use [3-5]. However, preoperative SSKI administration in GD plays a very important role in selected cases, because it is a very effective way for reaching euthyroidism quickly and better plan thyroid surgery, for example in patients, who do not tolerate thyreostatic therapy or in pregnant patients, who need to be operated during the second trimester.

Generally, iodine elixirs, up to 10 drops of saturated solution of potassium iodide (50 mg iodide per drop) are administered daily for seven to 10 days in combination with thyreostatic treatment. The ATA guidelines recommend 5-7 drops (0.25-0.35 mL) of Lugol's solution (8 mg iodide/drop) or 1-2 drops (0.05-0.1 mL) of SSKI (50 mg iodide/drop) three times daily mixed in water or juice for 10 days before surgery [1]. In the study of Lindner et al. [4], three drops of potassium iodide solution (30 mg iodide/drop) was administered twice daily for 13 days. This makes preoperative SSKI therapy a quite long and intensive inpatient treatment, requiring regular check of thyroid hormone levels. The solution must be prepared in the hospital pharmacy and, in our experience, it is not always quickly available.

Herein, we describe three selected cases in which concentrated potassium iodide 65 mg pills, as used in the event of a nuclear emergency with release of radioactive iodine, were administered for thyroid blocking preoperatively instead of SSKI.

## Case presentation

Case 1

A 32-year-old female patient in the 18th week of her second pregnancy was diagnosed with Graves’ disease in the 8th week [free triiodothyronine (fT3) 27.1 ng/L, thyrotropin receptor antibodies (TRAK) 25.4 U/L]. Because hyperthyroidism could not be controlled with 200 mg propylthiouracil daily, medication was changed to thiamazole, however fT3 remained elevated (increased from 5.7 to 10.9 ng/L) and thyroid volume increased from 30 to 80 ml in a short time. The medication was changed back to propylthiouracil 350 mg daily, however, hyperthyroidism was poorly controlled and the TRAKs remained elevated with over 40 U/L (normal < 1 U/L). Therefore, surgery during the second trimester was recommended after interdisciplinary case discussion in our endocrine board, consisting of an endocrinologist (M.F.), a nuclear medicine expert (M.S.) and an endocrine surgeon (C.C.) and after case discussion with the gynecology department.

The patient was admitted to the surgical ward and received in addition to propylthiouracil, 130 mg potassium iodide G.L. (two pills) daily as recommended by the German Federal Ministry of the Environment, Nature Conservation and Nuclear Safety [6] in the case of a nuclear incident. She tolerated this medication well.

Twenty-four hours after the first dose, fT3 dropped from 5.6 to 3.9 ng/L (normal range 2-4.4 ng/L). Forty-eight hours after admission and after having taken the second dose potassium iodide G.L. fT3 was 2.3 ng/L. Surgery was performed without complications.

The patient subsequently received L-Thyroxin and delivered a healthy female child five months later, whose thyroid hormones at birth were in the normal range.

Case 2

A 56-year-old female patient was diagnosed with Graves’ disease 12 years before presentation. She had been treated with propylthiouracil because of thionamide intolerance (manifesting with increased liver transaminases). She presented with thyroid storm (fT3 29.4 ng/L and TRAKs 25.4 U/L) and was treated with propylthiouracil 200 mg again and also beta-blockers. fT3 dropped to 8.6 ng/L but the patient complained of several symptoms including dizziness, muscle weakness, nausea, itching and muscle pain and asked for surgical removal of the thyroid gland. The indication was discussed in the interdisciplinary endocrine board and thyroidectomy was recommended by the endocrine tumor board.

She was admitted to the surgical department and received in addition to propylthiouracil, 130 mg potassium iodide (two pills) daily as recommended by the German Federal Ministry of the Environment, Nature Conservation and Nuclear Safety [6].

Twenty-four hours after the first dose fT3 had dropped to 4.5 ng/L, 48 h after admission and after two doses fT3 was 3.9 ng/L and surgery was performed without complications.

Postoperatively, the patient was treated with L-Thyroxin and is now in good health.

Case 3

A 20-year-old female patient with thyroid storm and fT3 >32.5 ng/L (normal range 2-4.4 ng/L) was presented from an external clinic. She received high dose thiamazole intravenously, betablockers and steroids and fT3 dropped to 1.7 ng/L within eight days. Symptoms improved, the patient was discharged with thiamazole 60 mg three times daily p.o. (by mouth). fT3 further improved from 9.4 to 7.1 ng/L, however the patient complained of dizziness, vomiting and skin rashes, especially in her hands and arms. Because of these side effects, the patient wish for a quick solution, and the remaining high TRAKs levels with 22.6 U/L (normal range <1.8 U/L), surgery was recommended after interdisciplinary discussion.

She was admitted to the surgical department and received 130 mg potassium iodide G.L. (two pills) daily as recommended by the German Federal Ministry of the Environment, Nature Conservation and Nuclear Safety in addition to thiamazole [6].

Twenty-four hours after the first dose fT3 dropped to 4.7 ng/L, and surgery was performed 48h after admission without any complications.

The patient received L-Thyroxin subsequently and is now in a good condition.

## Discussion

Although complication rates and especially intraoperative and postoperative bleeding risk in thyroid surgery for GD are not significantly elevated in the hands of experienced endocrine surgeons [3], and the recent literature shows no significant benefit in terms of operative time and complications [3-5], SSKI is helpful for restoring euthyroidism quickly, in order to perform thyroid surgery in selected cases. This is important in patients who do not tolerate thyreostatic treatment and ask for definitive treatment as well as in patients who need to undergo surgery within a certain time span, like pregnant women during the second trimester. SSKI, however, is not always easily available and must be ordered and prepared in the hospital pharmacy. Moreover, it must be administered for seven to 10 days in an inpatient setting and under regular check of thyroid hormone levels.

Potassium iodide G.L. pills (65 mg) instead are stored in every nuclear medicine department in Germany. The German Federal Ministry of the Environment, Nature Conservation and Nuclear Safety recommends 130 mg for adults and 65 mg for children in the event of a nuclear emergency with release of radioactive iodine as a single treatment.

In this case series, this treatment was extended to selected situations, in which euthyroidism had to be restored quickly. Treatment was administered in an inpatient setting and thyroid values were checked daily like for SSKI. However, euthyroidism could be reached faster: after 24 hours instead of a week. No adverse effects were observed.

Although this is only a series of three patients, our data suggest an easy approach without any side effects or complications to reach euthyroidism quickly in selected cases, when thyreostatic medication is not tolerated and early surgical treatment is necessary. However, like after SSKI, surgery should not be postponed in order to avoid rebound hyperthyroidism.

## Conclusions

Although a routine treatment of GD patients with potassium iodide cannot be recommended, it is still useful in several cases. Potassium iodide G.L. pills - as recommended by the German Federal Ministry of the Environment, Nature Conservation and Nuclear Safety in the event of a nuclear emergency with release of radioactive iodine - are an effective and easily available option to quickly restore euthyroidism preoperatively, if prompt thyroid surgery is mandatory and might be used instead of saturated solution of potassium iodide, which must be administered over several days in an inpatient setting.
